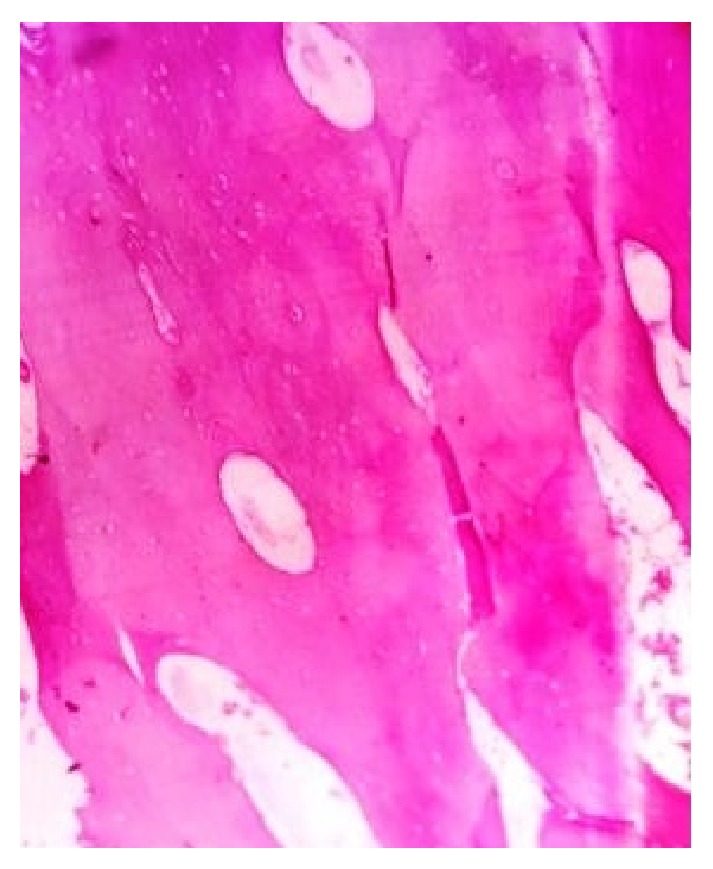# Solitary Peripheral Osteoma of the Angle of the Mandible

**DOI:** 10.1155/2015/430619

**Published:** 2015-09-01

**Authors:** Kapil Kshirsagar, Kalyani Bhate, Vivek Pawar, S. N. SanthoshKumar, Supriya Kheur, Shrikant Dusane

**Affiliations:** ^1^Department of Oral and Maxillofacial Surgery, Dr. D. Y. Patil Vidyapeeth Dr. D. Y. Patil Dental College and Hospital, Pimpri, Pune 411018, India; ^2^Department of Oral Pathology, Dr. D. Y. Patil Vidyapeeth Dr. D. Y. Patil Dental College and Hospital, Pimpri, Pune 411018, India; ^3^Department of Oral and Maxillofacial Surgery, Sinhgad Dental College and Hospital, Pune 411041, India

## Abstract

Solitary peripheral osteoma is a benign, slow-growing osteogenic tumor arising from craniofacial bones such as the sinus, temporal, or jaw bones but rarely originating from the mandible. Osteoma consists of compact or cancellous bone that may be of peripheral, central, or extraskeletal type. Peripheral osteoma arises from the periosteum and is commonly a unilateral, pedunculated mushroom-like mass. Solitary peripheral osteomas are characterized by well-defined, rounded, or oval radiopaque mass in the computed tomography. Although multiple osteomas of the jaws are a hallmark of Gardner's syndrome (familial adenomatous polyposis), nonsyndromic cases are typically solitary. Herein, we report a rare case of solitary peripheral osteoma of the angle of the mandible in a 27-year-old female with clinical, radiologic, and histopathologic findings.

## 1. Introduction

Solitary peripheral osteomas of facial bones are benign osteogenic tumors characterized by the proliferation of compact or cancellous bone. They originate from the craniomaxillofacial region such as temporal bones, sinuses, or maxilla or mandible. They are mostly seen occurring between 2nd and 5th decades, but they may be seen at any age. Three subtypes of osteomas are seen, peripheral (parosteal, periosteal, or exophytic), central (endosteal), and extraskeletal (osseous choristoma osteoma) [1, 2].

Histologically, osteomas may be of two types: (1) compact and (2) cancellous. Compact osteomas consist of dense, compact bone with a few marrow spaces, while cancellous osteoma is characterized by bony trabeculae and a fibrofatty marrow enclosing osteoblasts which resembles mature bone [3].

The most common location of the peripheral osteoma is the skull. However, lesions do seldom occur in the mandible, especially on the lingual aspect, inferior border, and body of the mandible and at the angle region [4].

Multiple osteomas of jaws are a hallmark of Gardner's syndrome (familial adenomatous polyposes), an autosomal dominant disease caused by a mutation in the APC tumor suppressor gene. In this syndrome, patient presents with multiple osteomas of the jaws along with multiple premalignant colorectal adenomas, which if left untreated progresses to colorectal carcinoma by middle age [3].

In this case report, we present a case of a solitary peripheral osteoma arising at the angle of the mandible with its clinicopathological and radiological findings along with differential diagnosis and treatment plan.

## 2. Case Presentation

A 27-year-old female patient reported to the Department of Oral and Maxillofacial Surgery with the chief complaint of hard swelling in the posterior region of the mandible. She gave the history that the swelling was first noticed about 2 years back, was insidious in onset, and gradually increased to the present size.

**Figure 1:**
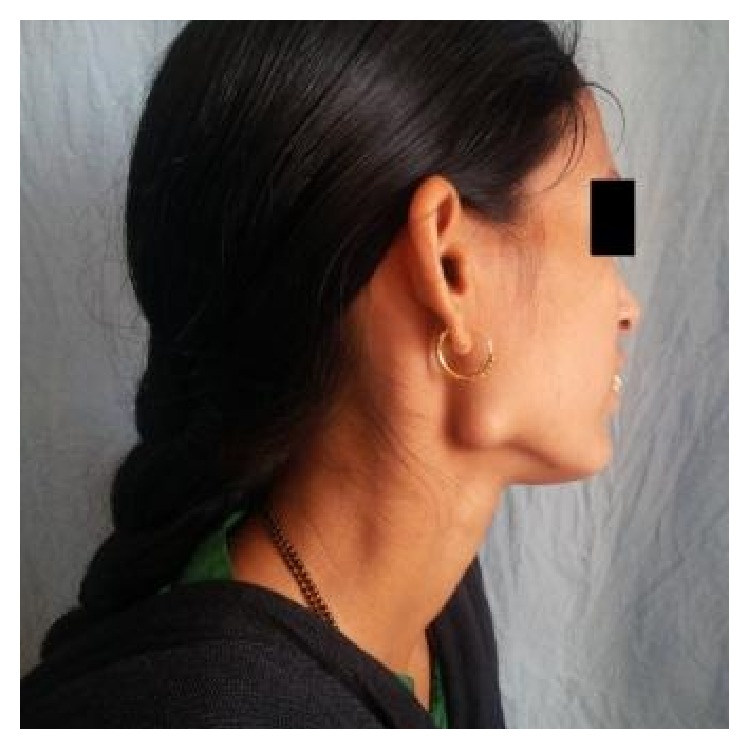


The swelling was not associated with any pain, discharge, fever, paresthesia, or difficulty in mastication. The patient did not give any history of trauma or infection in that region. On examination, a solitary, nonpulsatile, nontender, approximately 1.5 cm sized, bony hard mass was palpated at the right angle region of the mandible (Figure 1). The swelling was fixed to the underlying bone, with overlying skin normal in color and not attached to the underlying mass.

**Figure 2:**
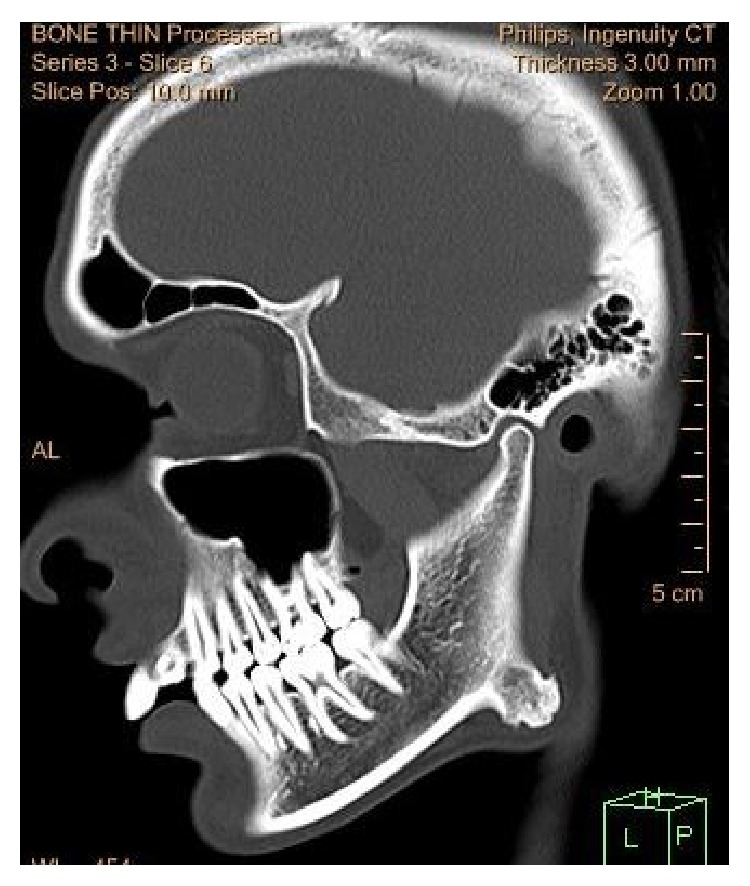


**Figure 3:**
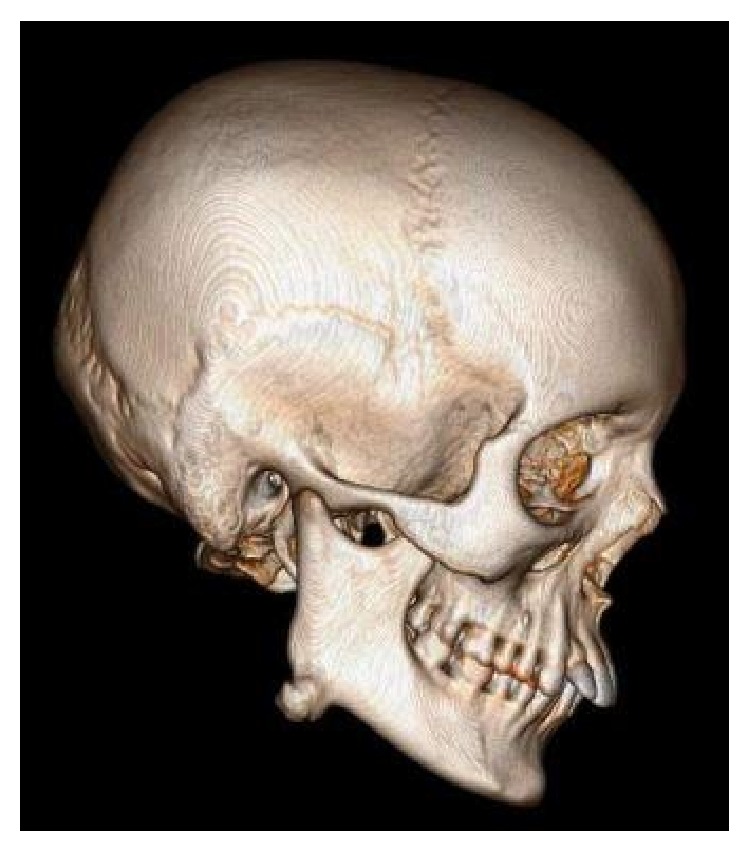


The computed tomography (CT) scan showed a well-circumscribed, hyperdense image with a lobulated surface located on the right angle of the mandible (Figure 2). The 3D reconstruction of the computed tomography image revealed a pedunculated homogeneous bony mass attached to the buccal cortex at the right angle of the mandible (Figure 3).

**Figure 4:**
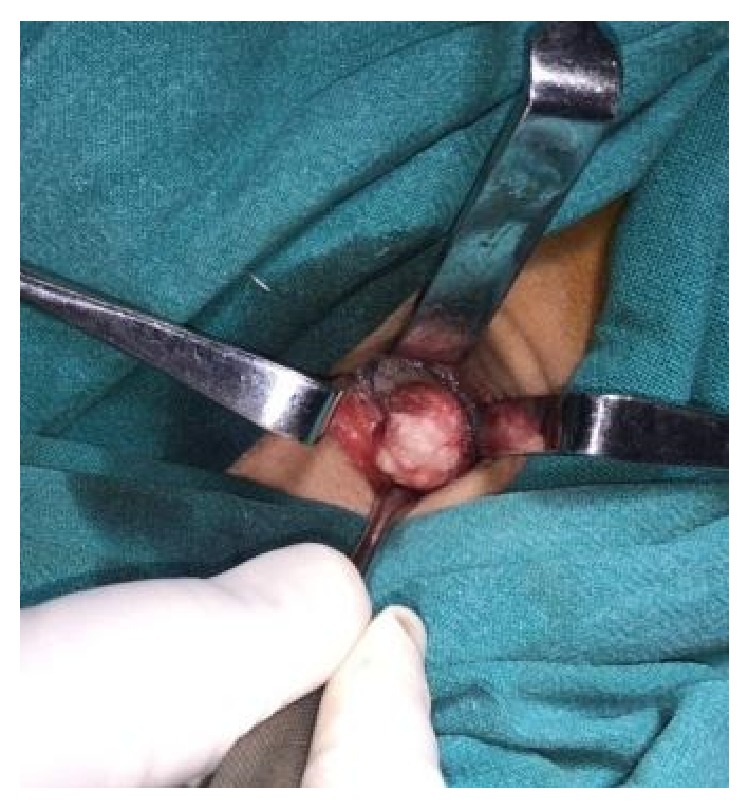


**Figure 5:**
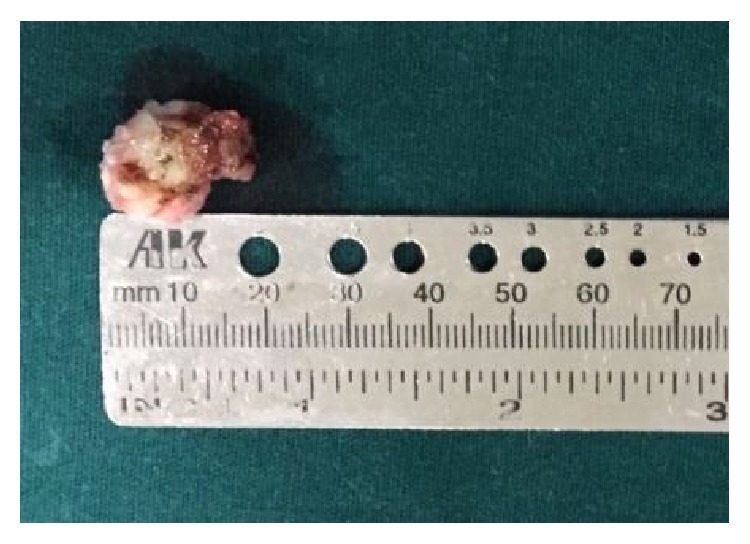


The lesion was completely excised using an extraoral approach under general anesthesia (Figure 4). Macroscopically, yellowish solid mass of about 1.5 cm in diameter was visualized (Figure 5). The surgical specimen was submitted for histopathological examination. Tissue specimen was fixed in 10% formaldehyde and then decalcified in 8% formic acid solution. They were processed routinely and the slides were stained with hematoxylin and eosin. Histopathological examination revealed areas of mature compact bone formation with haversian canals, lacunae with osteocytes, resting lines, and lamellae (Figure 6). These features confirmed the final histopathologic diagnosis to be compact osteoma.

## 3. Discussion

As solitary peripheral osteoma may be clinically silent for years without symptoms, it is usually diagnosed when it becomes enlarged or is incidentally discovered by radiological examination such as panoramic radiography or CT [1]. In our case, the lesion is single and silent and initially without any symptoms; however, it gradually enlarged over a period of 2 years to reach a size of 1.5 cm.

Radiographically, the presence of an oval, radiopaque, well-circumscribed mass attached by a broad base or pedicle to the affected cortical bone is a hallmark of peripheral osteomas [5]. Similarly, in the present case, the computed tomography (CT) scan showed a well-circumscribed, radioopaque mass with a broad base attached to the right angle of the mandible. The 3D reconstruction of the computed tomography image revealed a pedunculated homogeneous bony mass attached to the buccal cortex at the right angle of the mandible. Peripheral osteoma should be differentiated from several pathologic entities such as exostoses, erupting odontoma, and peripheral ossifying fibroma. Exostoses are hamartomas in preference to occur at the lingual [torus mandibularis] and buccal regions of the mandible, midline of hard palate [torus palatinus], and buccal and palatal region of the maxilla. These bony enlargements usually stop growing after puberty, being differentiated from osteomas [3, 6]. Usually odontomas are asymptomatic; however, pain and swelling are the most common symptoms when odontomas erupt, followed by malocclusion. An erupting odontoma presents with a welldefined radiopacity but also with a density that is greater than bone and equal to or greater than that of a tooth. A radiolucent halo, typically surrounded by a thin sclerotic line, surrounds the radiopacity [6, 7].

The pathogenesis of peripheral osteomas is unclear. Some investigators considered it a true neoplasm, while others classified it as a developmental anomaly. Peripheral osteomas exhibit continuous growth rather than growth cessation at adulthood, and this characteristic is the major feature distinguishing them from other bony exostoses supporting a neoplastic origin. In addition, the possibility of a reactive mechanism, triggered by trauma or infection, has also been suggested [8, 9]. Kaplan et al. suggested that many peripheral osteomas may be reactive lesions caused by trauma or muscle traction rather than neoplasm, because many peripheral osteomas are located on the lower border or buccal aspect of the mandible [8]. Close location of most of the peripheral osteomas with muscles such as masseter, medial pterygoid, and temporalis muscle suggests an etiology secondary to muscle traction [5].

Gardner's syndrome should be suspected with the detection of facial osteoma. Gardner's syndrome may present with rectal bleeding, diarrhea, and abdominal pain and may be characterized with colorectal polyposis, multiple osteoma, skin and soft tissue tumors, and multiple impacted or supernumerary teeth [10]. In our case, the lesion was solitary, there were no dental or skin anomalies, and the patient gave no history of pain or any intestinal complaints. Thus, Gardner's syndrome was not considered due to the absence of accompanying lesions.

A peripheral osteoma can be completely treated by surgical intervention. Surgery consists of removing the lesion at the base where it enters the cortical bone. Extraoral approach is preferred for peripheral osteomas that are located in the posterior region of the mandible. Recurrence after complete excision is very rare and malignant transformation has not been reported in the literature [11, 12]. In our case, the patient was reported with a lesion at the angle of the mandible and hence an extraoral approach was used.

Even though the solitary peripheral osteomas are benign lesions, surgical excision is recommended. Though recurrence is not commonly reported, radiographic follow-up every 6 months for 2-3 years with annual radiographs thereafter is advised.

## Figures and Tables

**Figure 6:**